# Dissecting genomic regions and candidate genes for pod borer resistance and component traits in pigeonpea minicore collection

**DOI:** 10.3389/fpls.2025.1630435

**Published:** 2025-08-29

**Authors:** Abhinav Moghiya, R.S. Munghate, Vinay Sharma, Suraj Prashad Mishra, Jagdish Jaba, Shailendra Singh Gaurav, Sunil S. Gangurde, Namita Dube, Sagar Krushnaji Rangari, Rajib Roychowdhury, Prakash Gangashetty, Hari Chand Sharma, Manish K. Pandey

**Affiliations:** ^1^ Center of Excellence in Genomics and Systems Biology (CEGSB), and Center for Pre-Breeding Research (CPBR), International Crops Research Institute for the Semi-Arid Tropics (ICRISAT), Hyderabad, India; ^2^ Department of Genetics and Plant Breeding, Chaudhary Charan Singh University (CCSU), Meerut, India

**Keywords:** marker-trait association, candidate gene discovery, genomic regions, mini-core collection, insect damage score, insect resistance

## Abstract

**Background:**

Pigeonpea is an important leguminous food crop primarily grown in tropical and subtropical regions of the world and is a rich source of high-quality protein. Biotic (weed, disease, and insect pests) and abiotic stresses have significantly reduced the production and productivity of pigeonpea. *Helicoverpa armigera*, also known as the pod borer, is a major pest in pigeonpea. A substantial investigation is needed to comprehend the genetic and genomic underpinnings of resistance to *H. armigera*. Genetic improvement by genomics-assisted breeding (GAB) is an effective approach for developing high-yielding *H. armigera*-resistant cultivars. Still, no genetic markers and genes linked to this key trait have been detected in pigeonpea. In this context, a set of 146 pigeonpea minicore accessions were evaluated for four *H. armigera*-resistant component traits, namely, pod borer resistance (PBR), days to 50% flowering (DF), days to maturity (DM), and grain yield (GY), for three consecutive seasons under field conditions.

**Results:**

Phenotypic data of pod borer resistance and component traits, along with the whole-genome resequencing (WGRS) data for 4,99,980 single nucleotide polymorphisms (SNPs), were utilised to perform multi-locus genome-wide association study (GWAS) analysis. Two models [settlement of MLM under progressively exclusive relationship (SUPER) and fixed and random model circulating probability unification (FarmCPU)] detected 14 significant marker–trait associations (MTAs) for PBR and three component traits. The MTAs with significant effect were mainly identified on chromosomes CcLG02, CcLG04, CcLG05, CcLG07, and CcLG11. These MTAs were subsequently delineated with key candidate genes associated with pod borer resistance (*probable carboxylesterase 15*, *microtubule-associated protein 5*, *FAR1-RELATED SEQUENCE*, and *omega-hydroxypalmitate O-feruloyl transferase 4*), days to maturity (*RING-H2 finger protein ATL7* and *leucine-rich repeat receptor-like protein kinase*), and grain yield (*secretory carrier-associated membrane protein* and *glutaredoxin-C5 chloroplastic*).

**Conclusion:**

These research findings reported significant MTAs and candidate genes associated with pod borer resistance and component traits. Further lab-based pod bioassay screening identified four minicore accessions, namely, ICP 10503, ICP 655, ICP 9691, and ICP 9655 (moderately resistant genotypes), showing the least damage rating and larval weight gain %, compared to the susceptible checks. After validating the significant MTAs, the associated SNP markers can be effectively utilised in indirect selection, which offers potential gains for such quantitative traits with low heritability and can improve insect management more sustainably. The significant MTAs, candidate genes, and resistant accessions reported in this study may be utilised for the development of pod borer-resistant pigeonpea varieties.

## 1 Introduction

Pigeonpea [*Cajanus cajan* (L.) Millsp.] is an important food legume crop in the arid and semi-arid regions of Asia and Africa. It is grown on 5.7 million hectares worldwide, with a production of 4.9 million tons (FAO, 2024). India, along with Malawi, Tanzania, Kenya, Uganda, and Myanmar, is a leading producer, contributing 78% of the global pigeonpea production. As one of the five major edible legumes, pigeonpea is used for edible purposes, animal feed, and firewood. It is an important source of protein, often used to supplement cereal-based diets (Kinhoégbè et al., 2022). Climate change presents a substantial risk to worldwide pigeonpea production, impacting both its nutritional quality and its ability to withstand various abiotic and biotic stresses. In India, pulses are vulnerable to approximately 150 insect pest species (Seetharamu et al., 2020), and globally, approximately 38 species of Lepidopteran insects harm pigeonpea (Shanower et al., 1999). Among the most damaging biotic stresses is the pod borer, *Helicoverpa armigera*, which severely affects crop growth and yield (Ghosh et al., 2017). Although pesticides can control the pod borer complex (PBC), the excessive use of chemical insecticides has resulted in insect resistance, secondary pest outbreaks, detrimental impacts on biodiversity, and negative environmental effects (Ambidi et al., 2021; Jaba et al., 2023). Therefore, developing pigeonpea varieties that are resistant to *H. armigera* is seen as the most effective solution to reduce pesticide use. Despite extensive screening of various pigeonpea genetic resources across Asia and Africa, no strong resistance against pod borer has been reported (Kambrekar, 2016). However, partial resistance has been reported in some cultivated genotypes, which have been utilised in pigeonpea breeding programs. While wild pigeonpea species confer higher pod borer resistance (PBR) compared to cultivated sources, transferring these resistance genes to cultivated varieties is limited to only a few wild species due to cross-incompatibility (Sharma, 2016; Singh et al., 2020). In earlier investigations, the International Crops Research Institute for the Semi-Arid Tropics (ICRISAT) minicore collection was screened, showing moderate resistance levels to the pod borer (Sharma et al., 2025, unpublished). These data have now been utilised for conducting a genome-wide association study (GWAS) and facilitating gene discovery.

The development and use of genomic tools can facilitate the selection of genotypes/breeding lines that are resistant to *H. armigera* using marker-assisted selection (MAS). However, there seems to be a lack of effort in identifying candidate genes and markers. Molecular markers are important for facilitating the transfer of insect-resistant genes to elite backgrounds, elucidating gene action, and minimising the negative effects of integrating undesirable genes from wild relatives due to linkage drag. Molecular breeding holds the potential to pyramid various sources of resistance that may not be efficiently selected by conventional breeding strategies due to phenotypic similarities, which can increase resistance levels and potentially develop resistant varieties (Sharma and Crouch, 2004). Recent breakthroughs in pigeonpea genomics research have resulted in the development of draft and telomere-to-telomere reference genomes (Varshney et al., 2012; Garg et al., 2022; Liu et al., 2024). Additionally, the accessibility of whole-genome sequencing (WGS) data (Varshney et al., 2017) and high-density Axiom *Cajanus* SNP arrays with 56K SNPs (Saxena et al., 2017) has significantly advanced genetic diversity, quantitative trait locus sequencing (QTL-seq), and genome-wide association study analysis. GWAS or association mapping has emerged as an important tool for identifying marker–trait associations (MTAs), candidate genes, and associated markers (Gudi et al., 2024; Sharma et al., 2024). Whole-genome resequencing (WGRS)-based GWAS is effective for identifying associated genomic regions and candidate genes related to specific traits in various legume species, including pigeonpea (Varshney et al., 2012; Xu et al., 2017; Kang et al., 2019). Recent studies have detected MTAs for flowering time (Kumar et al., 2022) and antioxidant properties (Megha et al., 2024). Similarly, meta-QTLs (MQTLs) were identified for agronomic traits, fertility restoration, disease resistance, and seed quality traits (Halladakeri et al., 2023). This investigation utilised multi-season phenotyping data generated on diverse minicore accessions to identify significant MTAs and candidate genes linked with pod borer resistance. We highlighted the importance of using various resistance sources against pod borer damage, emphasising the relationships between component traits (phenology and grain yield) and resistance levels. These findings facilitate the development of pigeonpea varieties exhibiting improved resistance to pod borer.

## 2 Materials and methods

### 2.1 Plant material

This investigation used 146 accessions from the International Crops Research Institute for the Semi-Arid Tropics (ICRISAT) minicore collection (Upadhyaya et al., 2006), along with two checks (resistant check ICPL 332WR and susceptible check ICPL 87). Seed material was procured from the ICRISAT Genebank (https://genebank.icrisat.org/IND/Passport?Crop=Pigeonpea&Location=Passport&mc=Yes).

### 2.2 Field experiment and phenotyping for pod borer resistance and component traits

Phenotypic screening of 146 accessions, including the susceptible check ICPL 87 and the resistant check ICPL 332WR, was performed using a randomised block design with three replicates during Rainy 2007 (S1), Rainy 2008 (S2), and Rainy 2009 (S3) at ICRISAT-Patancheru, Hyderabad. Each plot consisted of four rows with a row spacing of 30 cm and a plant spacing of 10 cm within each row. Plots were separated by a 1-m alley. Five randomly selected plants from each genotype and replication were tagged for recording observations on pod borer under natural infestation at the maturity stage. PBR was evaluated using a visual damage score on a scale of 1–9, where 1 indicates almost no damage (resistant) and 9 represents severe damage (highly susceptible) (**Supplementary Figure 1**), during the podding stage (Sujana et al., 2008). This was assessed alongside component traits such as days to 50% flowering (DF), days to maturity (DM), and grain yield (GY). DF and DM were recorded on a per-plant basis. The following pigeonpea descriptors (IBPGR and ICRISAT, 1993) were used to record the GY (g) per plant on five randomly selected representative plants per plot. The four best minicore accessions were subjected to a pod bioassay with artificial third-instar larvae (Ambidi et al., 2021).

### 2.3 Statistical analyses of phenotypic data

The statistical analysis of the phenotypic data was performed using RStudio version 4.3.1 (http://www.rstudio.com/). The “FactoMineR” package in R was used to perform Pearson’s correlation on replicated data from three seasons (Lê et al., 2008). The R package “phenotype” was used to calculate the best linear unbiased predictions (BLUPs) (Piepho et al., 2008).

### 2.4 DNA extraction and whole-genome resequencing

Genomic DNA was isolated from young leaves using the NucleoSpin^®^ 96 Plant II Kit (Macherey-Nagel), Düren, Nordrhein-Westfalen, Germany. The quality was assessed through 0.8% agarose gel electrophoresis, and the amount was quantified using a Qubit^®^ 2.0 fluorometer (Thermo Fisher Scientific Inc.,Waltham, Massachusetts, USA) (Pandey et al., 2020). Libraries with a 500-bp insert size were generated for all samples for WGRS, as detailed in Varshney et al. (2017). The fragments with insert sizes of approximately 500 bp were removed following separation on an agarose gel and then amplified by PCR. Furthermore, each library was subjected to sequencing on the Illumina HiSeq 2500 to generate paired-end reads. The raw reads were subjected to quality check using FastQC v0.11.8 and Trimmomatic v0.39; poor reads (Phred score < 30, read length < 35 bp) and adaptor exhibiting contamination were eliminated, resulting in high-quality reads. Furthermore, high-quality reads were aligned to the improved reference assembly (Cajca.Asha_v2.0) (Garg et al., 2022) using BWA version 0.5.9 (Li et al., 2009) with the standard parameters. SNP calling was conducted using GATK v.3.7 (McKenna et al., 2010). Biallelic SNPs exhibiting less than 20% missing calls and a minor allelic frequency cut-off of 5% and 50% heterozygosity were utilised for further analysis.

### 2.5 Linkage disequilibrium decay, GWAS analysis, and candidate gene identification

Linkage disequilibrium (LD) was analysed using TASSEL 5.0. The default parameters of PopLDdecay 3.4.2 were employed to calculate the decay of LD with physical distance. GWAS analysis was performed using 4,99,980 polymorphic SNPs and three seasons of pooled phenotyping data recorded on PBR and component traits. In our study, GWAS analysis was conducted employing four models—MLM, CMLM, fixed and random model circulating probability unification (FarmCPU), and settlement of MLM under progressively exclusive relationship (SUPER)—utilising R/GAPIT 4.3.1. The “Bonferroni correction” *p*-value threshold (<1.00004E−07) was implemented to remove false associations, and only MTAs with a phenotypic variance explained (PVE) >0% were considered (**Supplementary Table 1**). However, it was found that significant MTAs were only identified using the FarmCPU and SUPER models, which provided the most reliable and statistically significant results, so we considered the MTAs from these two models for downstream analysis. The physical position of significant MTAs with associated traits was used to mine candidate genes in these regions on the pigeonpea reference genome assembly v2.0 (Garg et al., 2022). We only considered genes where significant MTAs were located (genic and non-genic regions) based on variant annotation and the prediction of SNP effects using the open-source SNPEff-4.3T program.

## 3 Results

### 3.1 Phenotypic variation, heritability, and correlation for PBR and component traits

**Figure 1:**
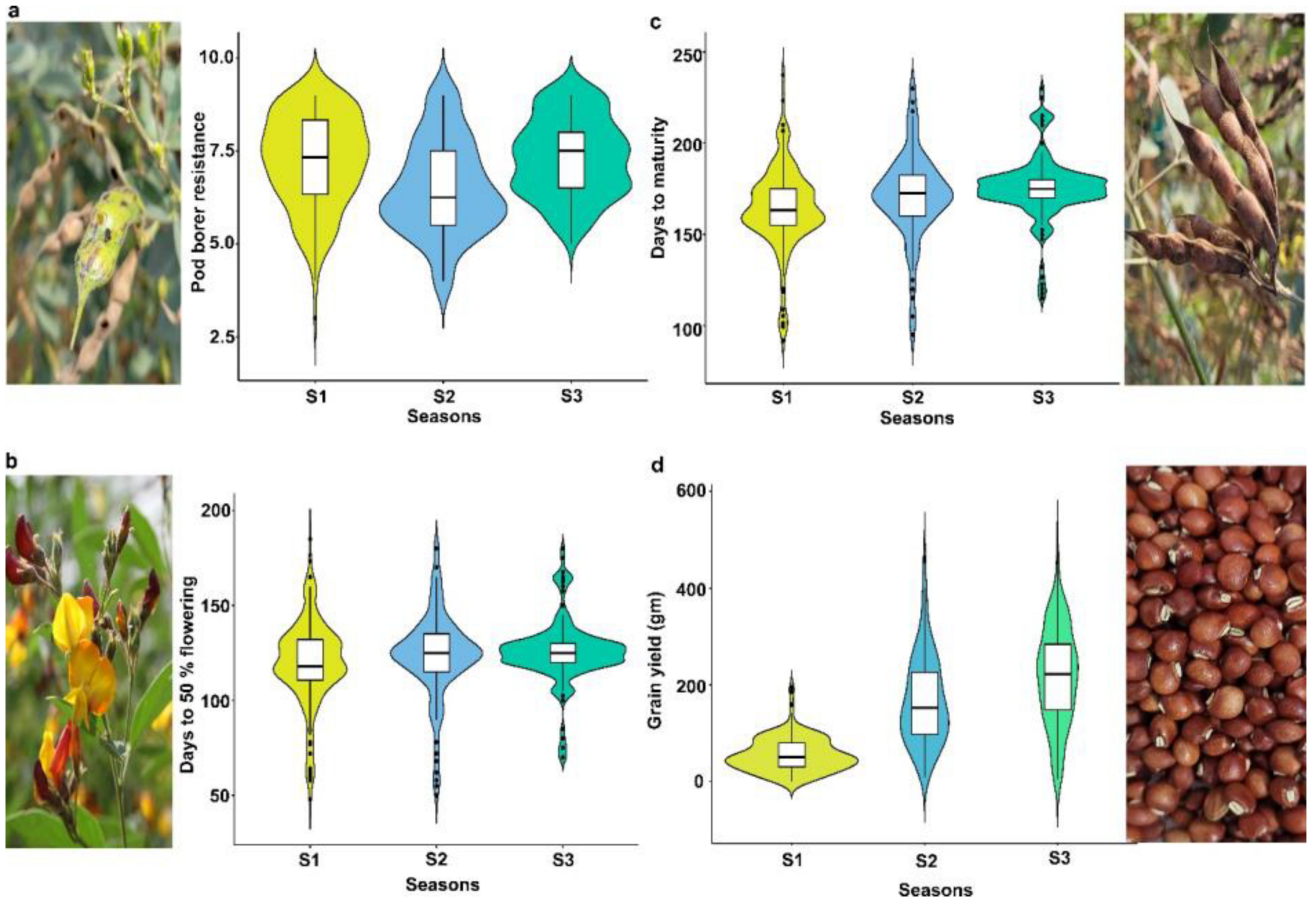
Phenotypic variation in minicore accessions for PBR and component traits. Violin plot showing variation for **(a)** pod borer resistance (PBR), **(b)** days to 50% flowering, **(c)** days to maturity, and **(d)** grain yield (g) traits consecutively evaluated for three seasons: S1, Rainy 2007; S2, Rainy 2008; and S3, Rainy 2009.

**Figure 2:**
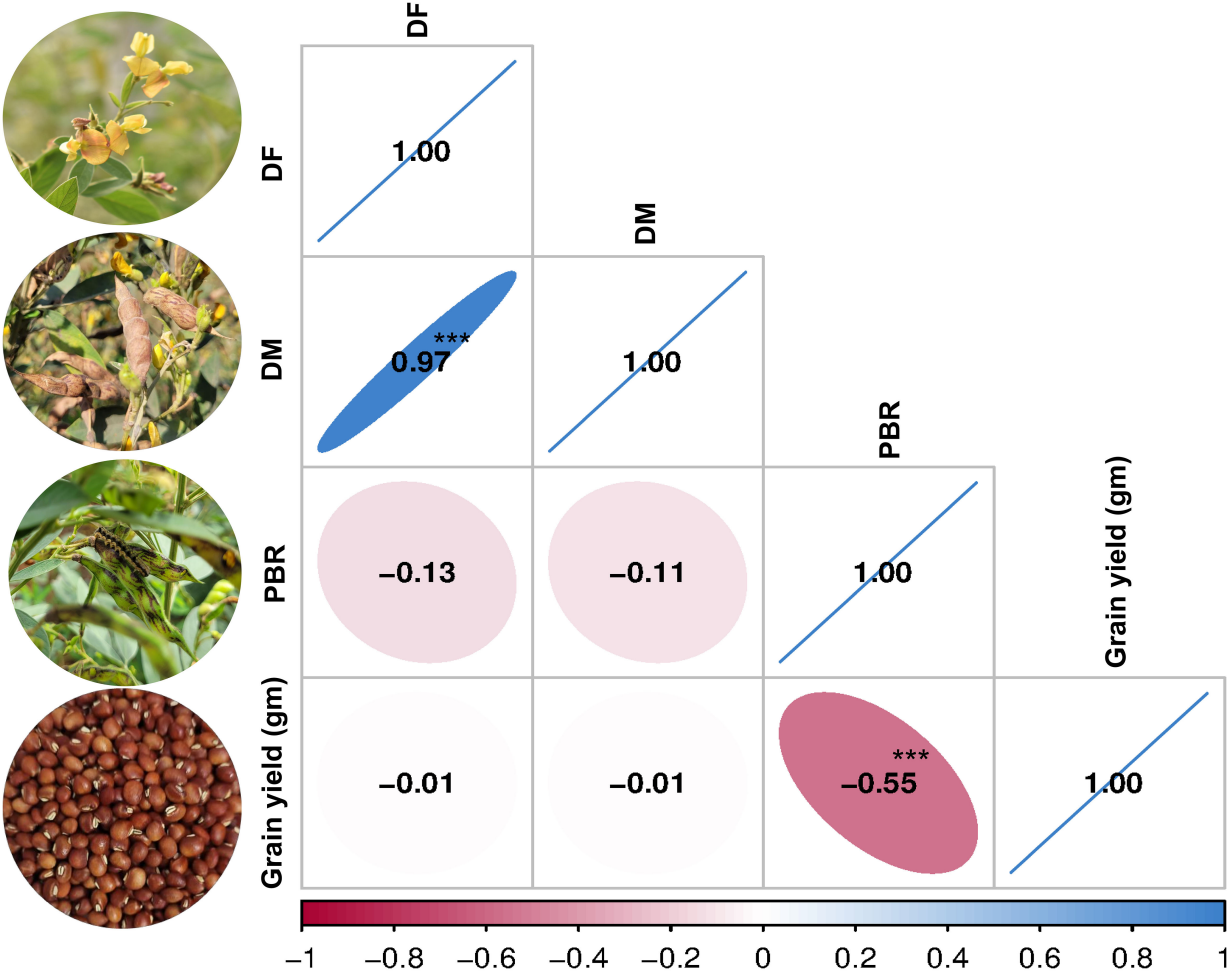
Pearson’s correlation matrix. Correlation between pod borer resistance (PBR) and component traits (***significant at 0.001 level).

Phenotypic evaluation for PBR and component traits showed significant variation among the minicore accessions. A symmetric distribution was observed for most of the traits (**Figure 1**). PBR score showed differences across seasons (3–9 in S1, 4–9 in S2, and 5–9 in S3). The average scores recorded were 7.3, 6.5, and 7.3 in S1, S2, and S3, respectively. Similarly, accessions revealed a wide range of variation for component traits: DF (48–185 in S1, 50–180 in S2, and 70–180 in S3), DM (90–245 in S1, 95–230 in S2, and 115–230 in S3), and GY (3–287 g plant^−1^ in S1, 8–463 g plant^−1^ in S2, and 6–481 g plant^−1^ in S3). Across seasons, all traits showed larger phenotypic coefficient of variation (PCV) and genotypic coefficient of variation (GCV) (>10%). Broad-sense heritability (h^2^) averaged 54% for PBR, 92% for DF, 96% for DM, and 55% for GY (**Table 1**). Following the replicated multi-season field evaluation results, 19 best lines were selected and screened using a lab-based pod bioassay. The gain % of the resistant check (ICPL332WR; score 6) compared to the susceptible check (ICPL 87; score 9) was assessed. Among the best lines, four—CP 10503, ICP 655, ICP 9691, and ICP 9655 (scoring between 4 and 5)—showed the least damage rating and low larval weight. Pearson’s correlation test was performed to determine the phenotypic correlation between PBR and component traits. A total of six possible correlations were observed, with three pairs (one positive and two negative). Correlation discussion revealed a strong positive correlation between DM and DF (r = 0.97), whereas GY and PBR had the highest negative correlation (r = −0.55), which was significant at the 0.001 level. Other correlations were not statistically significant (p > 0.05) (**Figure 2**).

**Table 1:** Mean, range, and variability components in minicore accessions for PBR and component traits across three seasons.

Traits	Seasons	Range	Grand mean	CD (5%)	SE (±)	GCV	PCV	h^2^ (%)
DF	S1	48–185	118.5	14.8	5.3	17.9	19.5	80
	S2	50–180	123.7	7.5	2.7	18.3	18.5	97
	S3	70–180	125.6	3	1.1	13.6	13.6	99
DM	S1	90–245	162.7	6.1	2.2	13.7	13.9	97
	S2	95–230	171.3	10.6	3.8	14	14.3	95
	S3	115–230	175.5	4	1.4	10.1	10.2	98
PBR	S1	3–9	7.3	1.8	0.7	13.3	20.6	42
	S2	4–9	6.5	1.6	0.6	16.7	21	63
	S3	5–9	7.3	1.5	0.5	12.3	16	58
GY (g)	S1	3–287	124.1	144	34.9	57.4	76.9	56
	S2	8–463	168.5	154.9	55.4	43.7	63.8	47
	S3	6–481	216.7	129	46.2	40.4	50.4	64

S1, Rainy 2007; S2, Rainy 2008; S3, Rainy 2009; DF, days to 50% flowering; DM, days to maturity; PBR, pod borer resistance; GY, grain yield; SE, standard error; CD, critical difference; GCV, genotypic coefficient of variation; PCV, phenotypic coefficient of variation; h^2^, broad-sense heritability.

### 3.2 LD decay and genome-wide association study analysis

**Figure 3:**
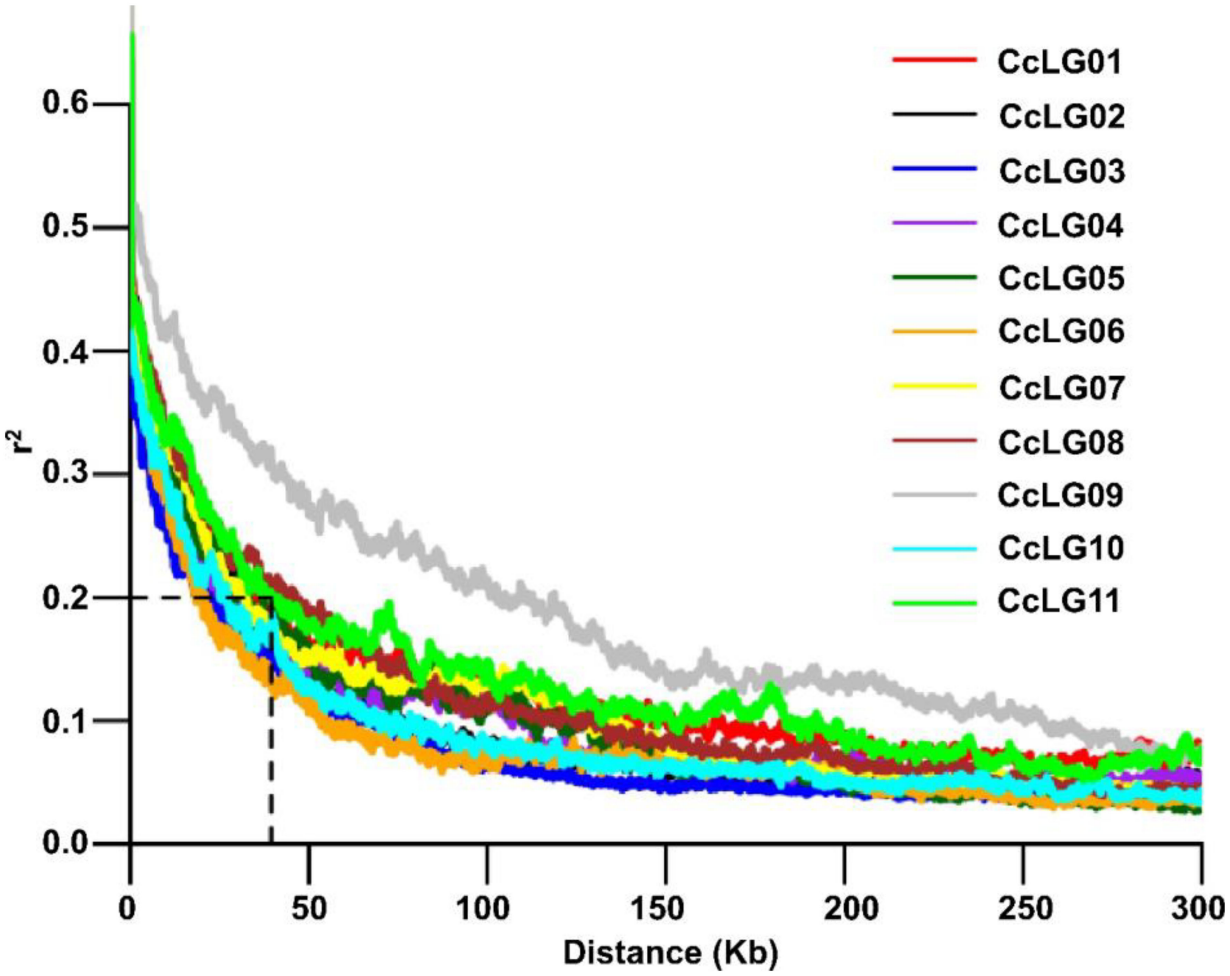
Estimated linkage disequilibrium decay (LD decay). LD decay for each chromosome, with r^2^ ~ 0.2 at 39.5 kb.

**Figure 4:**
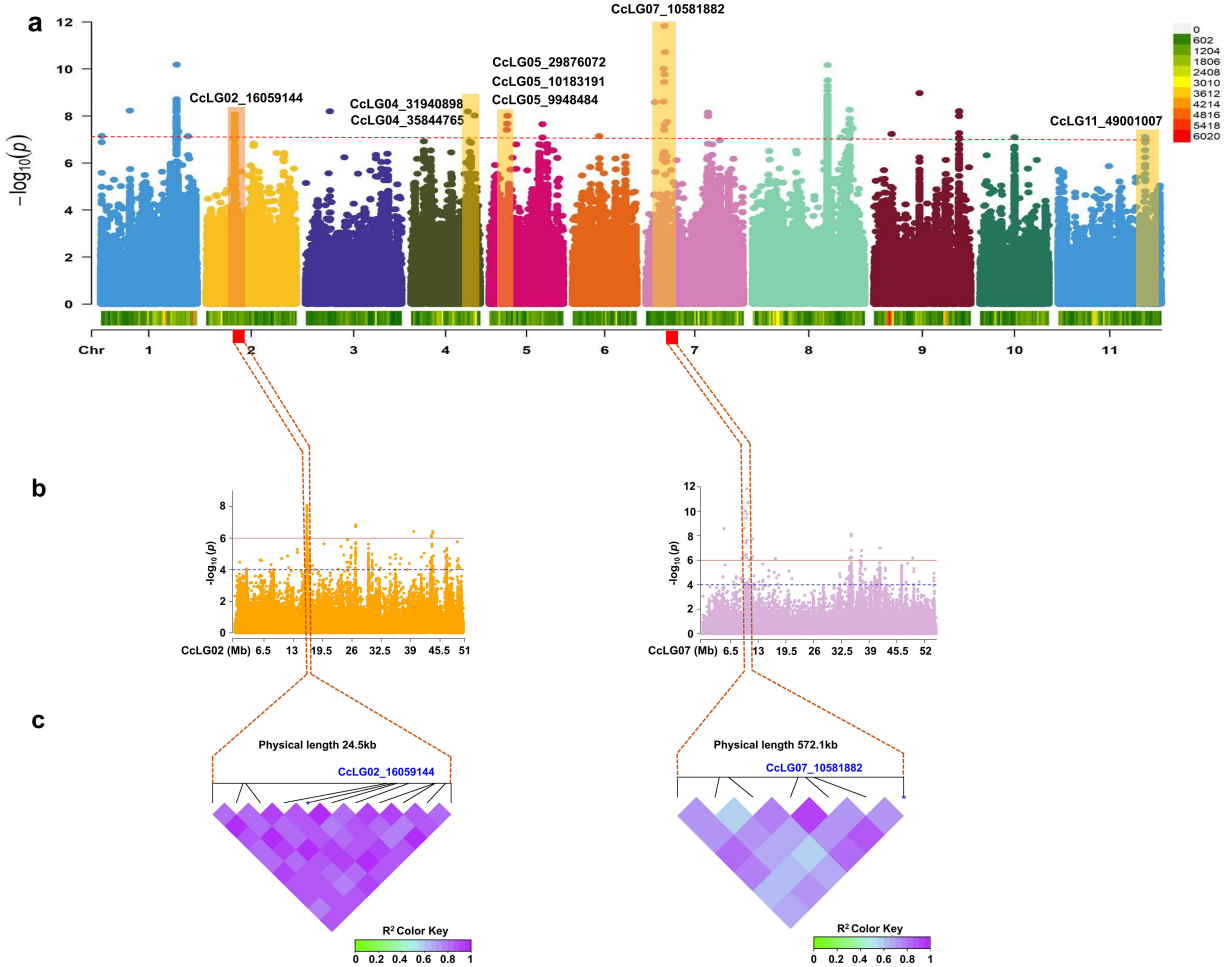
Genome-wide association study (GWAS) for pod borer resistance (PBR) trait. **(a)** Manhattan plot illustrating significant marker–trait association (MTA) for PBR trait. Only highly statistically significant MTAs at peak were considered. **(b)** Association for the significant SNPs on chromosomes CcLG02 and CcLG07. The interval of association was determined to lie between 24.5 kb downstream of the significant MTA (CcLG02_16059144) on CcLG02 and 572.1 kb downstream of the significant MTA (CcLG07_10581882) on CcLG07. **(c)** Linkage disequilibrium heatmaps for the association region for PBR on chromosomes CcLG02 and CcLG07. Bonferroni correction threshold of *p*-value (<1.00004E−07) was implemented to detect significant associations.

**Figure 5:**
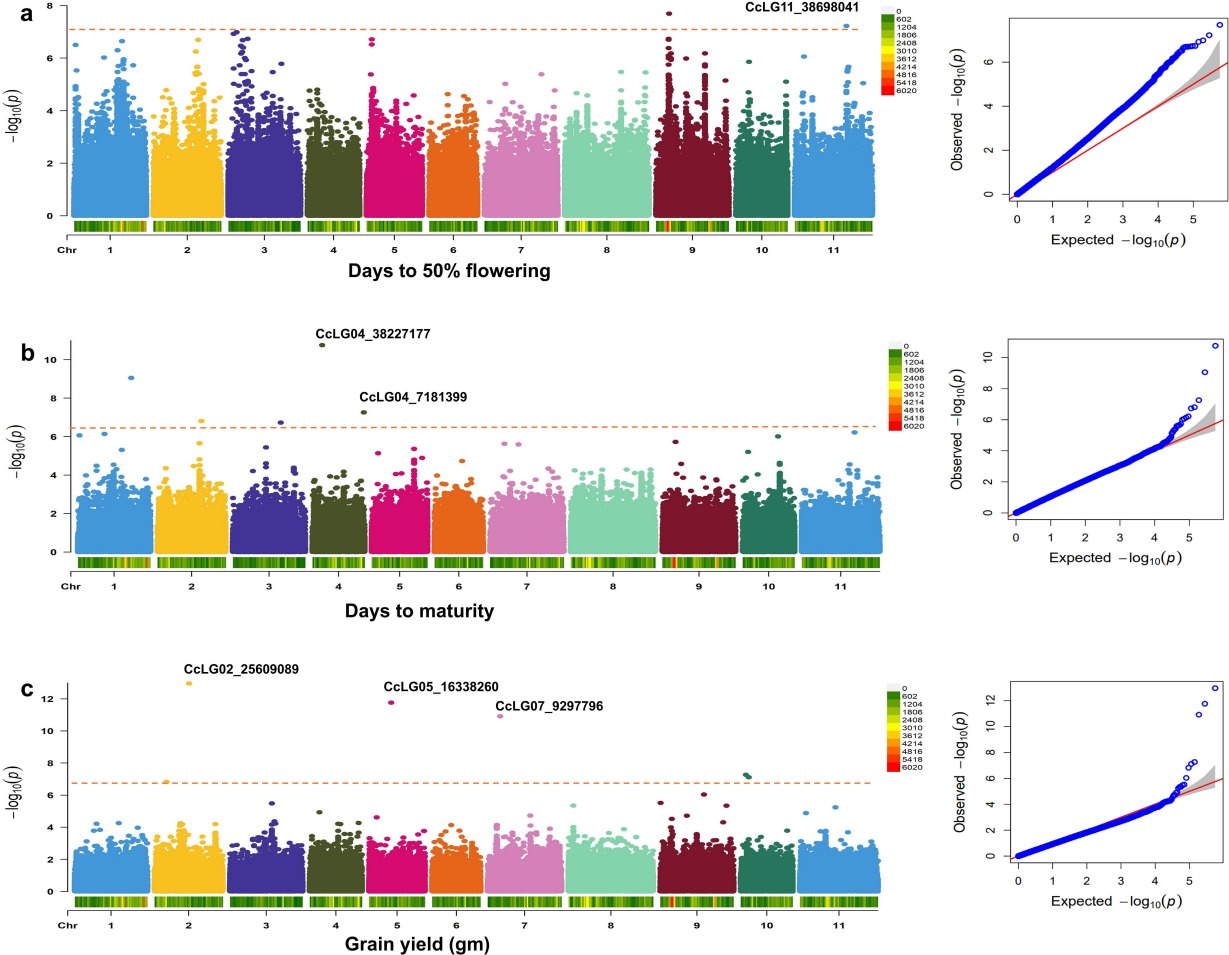
Genome-wide association study (GWAS) for component traits. Manhattan and quantile–quantile (QQ) plots for **(a)** days to 50% flowering, **(b)** days to maturity, and **(c)** grain yield. Bonferroni correction threshold of *p*-value (<1.00004E−07) was implemented to detect significant associations.

A total of 4,99,980 filtered SNP markers with a genotype call rate >0.80 and a minor allele frequency (MAF) of >5% were utilised for downstream analysis. The overall LD decay across the 11 chromosomes was 39.5 kbp on average (**Figure 3**). For the GWAS analysis, high-quality phenotyping data (three seasons pooled phenotyping data) for PBR and component traits were used along with 4,99,980 polymorphic SNPs. Two models (SUPER and FarmCPU) identified 14 significant MTAs (eight PBR, one DF, two DM, and three GY) for four traits, explaining 0.05%–28.1% phenotypic variation with a *p*-value range of 1.10E−13 to 9.66E−09 for pooled data (**Table 2**). Eight significant MTAs were identified for PBR on chromosomes CcLG02, CcLG04, CcLG05, CcLG07, and CcLG11, with PVE ranging from 0.05% to 5.57% (**Figure 4**). For DF, one MTA was detected (CcLG11_38698041) on the same chromosome (CcLG11) with PVE of 28.1%. On chromosome CcLG04, two MTAs (CcLG04_38227177 and CcLG04_7181399) for DM were detected, explaining 1.07%–2.74% PVE. Three MTAs were identified for GY on chromosomes CcLG02, CcLG05, and CcLG07, accounting for 0.49%–1.72% phenotypic variance (**Figure 5**). Based on pooled phenotyping data, a representative set of minicore accessions, exhibiting variability for PBR and component traits, were selected to *in silico* validate the SNPs associated with the significant MTAs. Among the 14 detected MTAs, five showed polymorphism, including two for PBR (CcLG04_35844765 and CcLG07_10581882) (**Supplementary Figure 2**) and three for component traits (one for DF, CcLG01_38698041; one for DM, CcLG04_7181399; and one for GY, CcLG04_25609089) in the minicore accessions (**Supplementary Figures 3–5**). These results indicate that the identified SNPs could be used to develop allele-specific markers for MAS, helping develop pigeonpea cultivars with improved resistance to *H. armigera*.

**Table 2:** Significant MTAs detected for PBR and component traits using multi-locus models with predictive gene variants and their functions.

Traits	Significant MTAs	Models	Chr	Position (bp)	*p*-Value	PVE (%)	Gene variants	Alleles	Gene ID	Functional annotation
PBR	CcLG02_16059144	SUPER	CcLG02	16,059,144	8.56E−09	1.52	DGV	G/A	*Cc_3152*	*Probable carboxylesterase 15*
PBR	CcLG04_31940898	SUPER	CcLG04	31,940,898	6.40E−09	1.30	DGV	T/C	*Cc_10409*	*Microtubule-associated protein 5*
PBR	CcLG04_35844765	SUPER	CcLG04	35,844,765	9.55E−09	5.57	UGV	G/A	*Cc_10514*	*Phospholipase D delta*
PBR	CcLG05_29876072	SUPER	CcLG05	29,876,072	2.21E−08	3.35	5′ UTR	A/T	*Cc_11847*	*Uncharacterized protein LOC100796483*
PBR	CcLG05_10183191	SUPER	CcLG05	10,183,191	9.66E−09	0.31	UGV	C/A	*Cc_11087*	*L-type lectin-domain containing receptor kinase IX.1*
PBR	CcLG05_9948484	SUPER	CcLG05	9,948,484	2.13E−08	0.15	UGV	C/A	*Cc_11074*	*Hypothetical protein GLYMA_14G106500*
PBR	CcLG07_10581882	SUPER	CcLG07	10,581,882	1.95E−11	0.05	UGV	G/A	*Cc_15562*	*Omega-hydroxypalmitate O-feruloyl transferase*
PBR	CcLG11_49001007	SUPER	CcLG11	49,001,007	7.86E−08	4.48	SV	T/C	*Cc_23491*	*FAR1-RELATED SEQUENCE 4*
DF	CcLG11_38698041	SUPER	CcLG011	38,698,041	5.96E−08	28.11	UGV	C/T	*Cc_23683*	*Hypothetical protein LR48_Vigan05g175700*
DM	CcLG04_38227177	FarmCPU	CcLG04	38,227,177	5.53E−08	1.07	UGV	G/T	*Cc_10599*	*RING-H2 finger protein ATL7*
DM	CcLG04_7181399	FarmCPU	CcLG04	7,181,399	1.74E−11	2.74	UGV	G/A	*Cc_9357*	*Leucine-rich repeat receptor-like protein kinase*
GY	CcLG02_25609089	FarmCPU	CcLG02	25,609,089	1.10E−13	0.49	UGV	C/T	*Cc_3417*	*Glutaredoxin-C5, chloroplastic*
GY	CcLG05_16338260	FarmCPU	CcLG05	16,338,260	1.75E−12	1.72	DGV	C/G	*Cc_11279*	*Secretory carrier-associated membrane protein*
GY	CcLG07_9297796	FarmCPU	CcLG07	9,297,796	1.22E−11	0.97	UGV	C/T	*Cc_15498*	*Hypothetical protein GLYMA_16G039300*

MTAs, marker–trait associations; Chr, chromosome; PBR, pod borer complex resistance; DF, days to flowering; DM, days to maturity; GY, grain yield; UGV, upstream gene variant; DGV, downstream gene variant; 5′ UTR, 5 prime UTR variant; SV, synonymous variant; MGV, missense gene variant; PVE, phenotypic variance explained; SUPER, settlement of MLM under progressively exclusive relationship; FarmCPU, fixed and random model circulating probability unification.

### 3.3 Putative genes associated with MTAs

The putative genes linked with the 14 significant MTAs identified for PBR and component traits were examined by analysing their location (genic and non-genic), effects, and functions (**Table 2**). Among these, 12 were detected in the intergenic regions, while one each was found in the exonic, 5′ UTR, and synonymous variant regions. Notably, MTA CcLG05_29876072, located in the 5′ UTR of gene (*Cc_11847*) on CcLG05 chromosome, was associated with PBR. Additionally, MTA CcLG11_49001007, located in the synonymous variant region of gene (*Cc_23491*) on CcLG11 chromosome, was also associated with PBR. Furthermore, the remaining significant MTAs associated with PBR, DF, DM, and GY were found in intergenic regions.

## 4 Discussion

Pigeonpea is a major grain legume that draws worldwide interest for its important contribution to nutritional and food security. However, *H. armigera* results in substantial losses in yield, posing a severe obstacle in pigeonpea cultivation (Sharma et al., 2022). Although significant breeding efforts have been made, the development of resistant varieties remains difficult due to the complex inheritance of resistance traits and the lack of genetic variation in cultivated germplasm (Volp et al., 2023; Karrem et al., 2025). Besides, transgenic approaches have potential legal issues and public acceptability concerns in India (Rakesh and Ghosh, 2024). Moreover, conventional pest management practices often lack sustainable solutions due to resistance to pesticides and environmental issues. Therefore, investigation of pigeonpea germplasm in the primary gene pool and crop wild relatives is an effective option since several *Cajanus* species exhibit higher resistance to *H. armigera* (Sharma et al., 2022; Singh et al., 2022). However, most cultivated genotypes showed low to moderate levels of resistance to *H. armigera*, evidenced by the screening of nearly 14,000 pigeonpea accessions (Reed and Lateef, 1990). Several investigations have reported that a few accessions of the wild progenitor of pigeonpea have exhibited high levels of resistance to *H. armigera* (Green et al., 2006; Sharma et al., 2009, 2022). It is important to understand that the trait is substantially influenced by genetic and environmental factors; therefore, relying solely on phenotypic screening for selection is insufficient. Furthermore, understanding the genetic basis of resistance to *H. armigera* can provide opportunities for developing resistant varieties. Our investigation on minicore accessions reported a broad range of variation in PBR and component traits. The h^2^ values of PBR, DF, DM, and GY were 54%, 92%, 96%, and 55%, respectively, indicating that a significant portion of the variation is attributable to distinct genotypes. The correlation analysis revealed a strong positive correlation between DM and DF, indicating that days to flowering could act as an index for maturity classification in pigeonpea. Previous investigations have shown similar findings for a correlation between DM and DF (Singh et al., 1995). Compared to other traits, GY and PBR had the strongest negative association (r = −0.55), which shows the highly influential nature of the trait. This indicates that a higher PBR score tends to be associated with lower GY, or vice versa. Furthermore, environmental factors such as excessive rainfall during sowing in S1, delayed planting, and variations in day and night temperatures throughout the reproductive stages likely contributed to the lower yield in S1, despite similar pod borer scores in other seasons. Previous studies have also reported that delayed sowing reduces yield in pigeonpea (Arunkumar et al., 2018).

MAS is an promising approach for accelerating the development of insect pest-resistant varieties. It facilitates the development of multi-trait resistant varieties by pyramiding different resistance genes to target insects, which is not possible with traditional breeding due to similar expression of phenotype (Sharma and Crouch, 2004). Utilising WGRS data along with precise phenotypic variability could help identify accessions with rare variants that may be potentially linked with important traits, such as resistance to *H. armigera*. In GWAS, determining the pattern of LD is important since it influences the resolution and magnitude of the association analysis. Our analysis showed an average LD decay at 39.5 kbp. The rapid LD decay indicates a minimal extent of long-range LD among the minicore accessions. A previous study reported genome-wide LD decay at 118 kb (Megha et al., 2024). GWAS minimises the two primary constraints of traditional linkage mapping, such as limited allelic diversity and insufficient genetic resolution (Huang and Han, 2014). Due to its high resolution and low cost in sequencing/genotyping, GWAS analysis has successfully dissected important traits in pigeonpea, including flowering-related traits (Kumar et al., 2022) and antioxidant activity (Megha et al., 2024). The main concern for GWAS is to minimise false positives, mostly due to population structure and familial relatedness (Kaler et al., 2020). Although single-locus models overcome this issue by including the two confounding factors as covariates, over-fitting in a model usually leads to false negatives, which could eliminate valuable loci (Price et al., 2006). In this context, multi-locus models provide an alternative way for reducing false negatives (Zhang et al., 2019). Multi-locus GWAS models, such as the SUPER and FarmCPU methods, improve statistical power but minimise false positives. The SUPER model offers greater computational power and requires less computing than earlier models. However, it extracts a small number of SNPs termed pseudo-quantitative trait nucleotide (QTN) to determine kinship (Wang et al., 2014). Moreover, “FarmCPU” is a novel multi-locus model that is computationally powerful and efficiently controls false negatives and false positives. Two multi-locus methods (SUPER and FarmCPU) were included in the current investigation to identify significant MTAs for PBR and component traits. GWAS analysis identified 14 significant MTAs linked to four traits, including eight for PBR, three for GY, two for DM, and one for DF. For the DF trait, one MTA was detected on chromosome CcLG11, accounting for the highest phenotypic variation of 28.1%. Most of the identified MTAs exhibited smaller phenotypic variation % and lower *p*-values. This finding suggests that these traits are controlled by multiple genes with minor effects, reflecting complex genetic architecture, and are also influenced by environmental factors. The statistical power of association mapping could be substantially improved by increasing the population size (Liu et al., 2021). The MTAs detected for PBR and component traits in our study were not reported previously and seem to indicate novel genetic loci in pigeonpea. Thus, the SNPs associated with MTAs offer the possibility of additional validation in diverse collections and may be utilised for early generation selection in breeding programs.

A total of 14 significant MTAs for four traits were detected and linked with putative genes. One MTA for PBR was found on chromosome 2 (CcLG02_16059144) linked to the *Cc_3152* gene encoding a *probable carboxylesterase 15* enzyme that catalyses the conversion of carboxylic esters and water into alcohol and carboxylate. In plants, it is involved in defence, development, and secondary metabolism (Palayam et al., 2024). In tobacco, this gene (NbCXE) is involved in host defence responses against Tobacco mosaic virus (TMV) infection (Guo and Wong, 2020). Similarly, another MTA (CcLG04_31940898) was identified for PBR encoding *microtubule-associated protein 5*, which plays a key role in cell division, cell proliferation, and cell morphology. In *Arabidopsis*, the microtubule-binding protein (*TGNap1*) facilitates the secretion of antimicrobial proteins, important for defence against phytopathogens (Bhandari et al., 2023). The MTA detected for PBR (CcLG11_49001007) in the exonic region of gene *Cc_23491*, which encodes *FAR1-RELATED SEQUENCE*, is a light signalling factor pair with *FAR-RED ELONGATED HYPOCOTYL* 3 to regulate plant immunity by integrating chlorophyll biosynthesis with the salicylic acid (SA) signalling pathway in *Arabidopsis* (Wang et al., 2015). For PBR, three more MTAs were detected and associated with *Cc_10514*, *Cc_11087*, and *Cc_15562*. Gene *Cc_10514* encodes *phospholipase D delta*, a protein that is involved in basal defence and non-host resistance to powdery mildew fungi in *Arabidopsis* (Pinosa et al., 2013). *Cc_11087* encodes an L-type lectin domain-containing receptor kinase IX, involved in self/non-self-surveillance and plant resistance. The homologues of these receptors in *Nicotiana benthamiana* and *Solanum lycopersicum* have the same role in defence against *Phytophthora* (Wang et al., 2015), and an MTA identified for PBR on chromosome 7 (CcLG07_10581882) associated with the *Cc_15562* gene encoding *omega-hydroxypalmitate O-feruloyl transferase* has a role in suberin biosynthesis. Suberin is synthesised in plant wound tissues to prevent pathogen infection (Molina et al., 2009). For PBR, two MTAs (CcLG05_29876072 and CcLG05_9948484) were detected in exonic (*Cc_11087* gene) and intergenic (*Cc_11074* gene) regions. These were predicted to encode an *uncharacterized protein* and a *hypothetical protein GLYMA_1*.

The MTA (CcLG04_38227177) identified for DM lies in the *Cc_10599* gene, which encodes a *RING-H2 finger protein*, which is important for seed development in *Arabidopsis* (Xu and Quinn Li, 2003). The MTA (CcLG04_7181399), identified for DM, is associated with the *Ca_00148* gene, which encodes a *leucine-rich repeat receptor-like protein kinase*. This protein is an important membrane-bound regulator of abscisic acid (ABA) early signalling in *Arabidopsis*, and ABA is involved in seed maturation (Osakabe et al., 2005). For GY, the *CcLG05_25609089* MTA was associated with the *Cc_3417* gene. *Cc_3417* encodes a *glutaredoxin-C5 chloroplastic protein*. Overexpression of a *CPYC-type glutaredoxin* was shown to increase grain weight in rice (Liu et al., 2019). Another MTA (CcLG05_16338260) is present in the intergenic region of the *Cc_11279* gene, encoding for the *secretory carrier-associated membrane protein*. Karnik et al. (2013) demonstrated that secretory carrier membrane proteins (SCAMPs) are involved in the secretion of defence proteins, including protease inhibitors and toxins, in *Arabidopsis thaliana*. These proteins have been shown to inhibit insect feeding or growth. However, further validation of the identified MTAs is required across varying genetic backgrounds. This provides deeper insights into the genetic control of resistance mechanisms, along with the potential to develop effective markers (Thakur et al., 2025). Additionally, gene editing innovations offer promising tools for validating and modifying the candidate genes identified by GWAS. It enables the precise knock-in or knockout of specific genes, providing clear evidence of their role in resistance. The integration of detected genes and SNPs associated with MTAs through molecular breeding or genetic modification could provide an effective approach for developing *H. armigera*-resistant cultivars.

## 5 Conclusion

Pod borer, *H. armigera*, is one of the most damaging pests in pigeonpea production. Various methods have been employed for controlling this pest, but have exhibited limited success. Phenotypic data on PBR and component traits, along with genotypic data from the WGRS, were used to identify 14 significant MTAs. These significant MTAs had 0.05%–28.1% phenotypic variation with a *p*-value range of 1.10E−13 to 9.66E−09. MTA for DF (CcLG11_38698041) on chromosome (CcLG11) had the highest PVE of 28.1%. Furthermore, we identified that important genes that encode *probable carboxylesterase 15* (*Cc_3152*), *microtubule-associated protein 5* (*Cc_10409*), and *FAR1-RELATED SEQUENCE* (*Cc_23491*) have been associated with plant defence responses and the regulation of plant immunity. These putative genes can be helpful for the identification of molecular targets, providing insight into the biological pathways that underlie the traits of interest and facilitating understanding of the genetic basis of complex traits. The importance of these genomic regions for future studies will help to understand the *H. armigera*-resistant mechanism, along with finding functional markers. Notably, further lab-based pod bioassay screening identified four minicore accessions—ICP 10503, ICP 655, ICP 9691, and ICP 9655—which showed moderate resistance. The resistant genotypes, significant MTAs, and putative genes identified in this investigation have the potential to be utilised in the development of pod borer-resistant pigeonpea cultivars.

## Data Availability

The datasets presented in this study can be found in online repositories. The names of the repository/repositories and accession number(s) can be found in the article/**Supplementary Material**.
